# Development of a Health-Related Quality of Life Questionnaire (HRQL) for patients with Extremity Soft Tissue Infections (ESTI)

**DOI:** 10.1186/1471-2334-6-148

**Published:** 2006-10-11

**Authors:** Aric J Storck, Kevin B Laupland, Ronald R Read, Manuel W Mah, John M Gill, Deborah Nevett, Thomas J Louie

**Affiliations:** 1 Department of Emergency Medicine, Calgary Health Region, Calgary, Alberta, Canada; 2 Department of Medicine, Calgary Health Region and University of Calgary, Calgary Alberta, Canada; 3 Department of Community Health Sciences, University of Calgary, Calgary, Alberta, Canada; 4 Home Parenteral Therapy Program, Peter Lougheed Centre, Calgary, Alberta, Canada

## Abstract

**Background:**

Past clinical trials of antimicrobial treatment in soft tissue infections have focused on non-standardized clinical and physiological outcome variables, and have not considered the subjective experience of patients. The objective of this study was to develop a health-related quality of life questionnaire (HRQL) for patients with extremity soft tissue infections (ESTI) for future use in clinical trials.

**Methods:**

The design of this study followed published guidelines and included item generation, item reduction, and questionnaire preparation. Study subjects were consenting English-speaking adults with acute ESTI requiring prescription of at least two days of outpatient intravenous antibiotic therapy.

**Results:**

A list of 49 items that adversely impact the quality of life of patients with ESTI was generated by literature review, informal health professional feedback, and semi-structured interviews with twenty patients. A listing of these items was then administered to 95 patients to determine their relative importance on quality of life. A questionnaire was prepared that included the twenty most important items with a 5-point Likert scale response. Questionnaire domains included physical symptoms, problems performing their activities of daily living, impairment of their emotional functioning, and difficulties in their social interactions as related to their ESTI. The final questionnaire was pre-tested on a further ten patients and was named the ESTI-Score.

**Conclusion:**

The ESTI-Score is a novel instrument designed to quantify the impact of ESTI on quality of life. Future study is required to determine its validity and responsiveness before use as an outcome measure in clinical trials.

## Background

Soft tissue infections (STI) are among the most common infectious diseases requiring antibiotic treatment [1,2]. Despite their prevalence and importance, the optimal treatment of STI's has not been well defined and considerable practice variation exists with respect to their management. Although a wide variety of different antibiotic classes, doses, durations, and methods of delivery have been compared in clinical trials, few studies have shown superiority of a given treatment [3]. While this may represent true clinical equipoise, it may also at least in part reflect the insensitivity of outcome measures used. These most commonly are non-standardized, disease-oriented, physician assessments that may not adequately assess the effect of therapy on the patient's subjective treatment responses.

The use of Health Related Quality of Life (HRQL) scales have been developed and demonstrated to be useful and valid primary outcome measures in clinical trials in a number of diseases not limited to asthma, rhinoconjunctivitis, polycystic ovarian syndrome, and sleep apnea [4-8]. Although quality of life measures have been developed for chronic skin diseases, no HRQL clinical instrument has been previously developed for acute extremity soft tissue infections (ESTI) [9,10]. The objective of this study was therefore to develop a disease-specific instrument to measure the HRQL in patients with ESTI for future use in clinical trials.

## Methods

The study methodology followed previously published guidelines for development of HRQL instruments [11,12]. This process included item generation, item reduction, questionnaire preparation, and questionnaire pre-testing. The study protocol was reviewed and approved by the Conjoint Health Research Ethics Board of the University of Calgary. All subjects provided written informed consent prior to participation. In addition to questionnaire specific data, information was also collected on patient demographics, co-morbidities, and the treatments prescribed.

### Study subjects

Patients with ESTI were enrolled from the Home Parenteral Therapy Program (HPTP) clinic at the Peter Lougheed Centre, Calgary, Alberta, Canada [13]. The HPTP is an ambulatory clinic that serves patients with a variety of infectious diseases requiring parenteral antimicrobial therapy. It is attended by fully certified infectious disease specialists. Between November 2003 and May 2005 a convenience sample of patients was screened for inclusion in the study. Patients were eligible for the study if they met all of the following: 1) age at least eighteen at the time of enrollment, 2) a diagnosis of ESTI made by an HPTP physician, and 3) judged to require two or more days of parenteral antibiotics by the HPTP physician. Patients were excluded if they had any of the following: 1) a chronic ulcer with no evidence of acute infection, 2) inability to speak and read English, 3) they were expected to have poor adherence to the study protocol, or 4) they were ineligible for the outpatient HPTP clinic (eg: admitted to hospital). Patients were otherwise included in the study irrespective of the severity of their illness, their co-morbidities, or specific treatment regimen prescribed.

### Item generation

The goal of this phase was to identify all possible items that might adversely impact the quality of life of patients with ESTI. These items were identified using four standard methods: 1) a review of the published literature on the clinical features of soft tissue infections, 2) a review of HRQL scales in other medical conditions, 3) semi-structured interviews with a group of patients, and 4) the collective experience of the study investigators and clinic staff. The patient interviews were performed by trained research nurses and took approximately 30 minutes. The interview used open-ended questions to identify all possible areas of dysfunction (including physical symptoms, activities of daily living, social and emotional well being) that might be adversely affected by their ESTI.

### Item reduction

The purpose of this phase was to identify the most important items for patients with ESTI. Study participants independently completed a questionnaire containing the items identified in the item generation phase. They were asked to identify which of the items were problems for them. For items identified as being a problem, they were asked to rate how important it was on a 5-point Likert scale (ranging 1, not very important, to 5, extremely important). The overall importance of each item was determined by calculating its frequency importance product (FIP). This was done by multiplying the frequency that a particular item was reported as a problem by the mean importance assigned to it [8].

### Final questionnaire preparation

The twenty items with the highest FIPs were incorporated into the final questionnaire. It was pre-tested on another group of consenting patients to ensure that it was free of grammatical errors and that individual items and the overall format were easily understood.

### Statistical considerations

Sample sizes were chosen *a priori *to be 20 patients for item generation and 100 patients for item reduction based on the guidelines of Kirshner and Guyatt [12]. All statistical analysis was performed using Stata 8.0 (StataCorp, College Station, Texas). Medians with interquartile ranges (IQR) were used to describe non-normally distributed variables and means with standard deviations (SD) for normally distributed variables. Group means were compared using Fisher's t-test. A p-value <0.05 was deemed to represent statistical significance.

## Results

### Item generation

Through interviews with twenty consenting patients with ESTI, a review of the relevant literature, and the collective experience of the study investigators, a list of 49 items was generated for inclusion in the item reduction questionnaire and these are shown in Table 1.

**Table 1:** Items included in the Item Reduction Questionnaire. Overall importance of each item as measured by the Frequency Importance Product.

Item	Frequency*	*Mean importance+*	*Overall importance++*
***Symptoms***			
**Pain**	0.95	3.69	3.49
Fever	0.42	3.23	1.36
Chills	0.51	3.06	1.55
Sweating	0.43	2.84	1.23
Loss of appetite	0.46	3.07	1.42
Nausea	0.27	2.63	0.72
Numbness	0.40	2.74	1.10
**Swelling**	0.92	3.88	3.56
Blistering	0.28	3.28	0.93
Fatigue	0.54	3.40	1.83
**Trouble moving**	0.75	3.47	2.59
**Stiffness**	0.60	3.28	1.97
**Soreness**	0.92	3.76	3.44
**Pressure**	0.66	3.43	2.28
**Throbbing**	0.61	3.36	2.05
Streaking	0.20	2.81	0.56
Discomfort in a distant body part	0.40	3.36	1.34
Discomfort caused by treatment of your infection	0.33	2.74	0.89
			
***Daily Functioning***			
**Doing your job**	0.79	3.96	3.13
**Walking**	0.59	3.48	2.05
**Bathing**	0.62	3.45	2.14
Using the toilet	0.38	2.97	1.13
Driving	0.51	3.27	1.65
**Changing clothes**	0.64	2.98	1.92
Hobbies	0.51	3.10	1.57
**Earning an income**	0.54	4.08	2.19
**Exercising**	0.66	3.20	2.12
**Falling asleep**	0.62	3.53	2.19
Traveling	0.38	3.03	1.15
			
***Emotional Functioning***			
**Frustrated**	0.68	3.46	2.37
Impatient	0.46	3.33	1.54
Irritable	0.57	3.09	1.76
Angry	0.28	3.27	0.93
**Disappointed**	0.61	3.18	1.94
Afraid	0.47	3.27	1.55
**Annoyed**	0.55	3.50	1.92
Embarrassed	0.31	2.77	0.85
Miserable	0.34	3.26	1.10
Depressed	0.28	2.67	0.76
Sad	0.34	2.75	0.93
Stressed	0.51	2.98	1.51
**Exhausted**	0.58	3.38	1.96
**Inconvenienced**	0.75	3.41	2.55
			
***Social Interactions***			
Difficulty socializing because your infection is "disfiguring"	0.19	2.57	0.49
Difficulty getting out of the house	0.41	3.21	1.32
Less desire to socialize than usual	0.40	3.11	1.24
**You are inconveniencing your friends and family**	0.56	3.37	1.88
Difficulty being physically intimate with your partner	0.36	3.56	1.27
Difficult being emotionally intimate with your partner	0.24	3.33	0.81

*Frequency of study participants reporting item as a problem (maximum = 1.0)

+Mean importance assigned to item if reported as a problem (maximum = 5.0)

++Frequency importance product (frequency × mean importance) (maximum = 5.0)

NB: The twenty highest ranking items are in bold font and were included in the final questionnaire and calculation of the ESTI score.

### Item reduction

Ninety-five patients were ultimately included in this stage. One hundred and three patients were initially enrolled but eight patients were subsequently excluded because they did not maintain enrollment criteria. In six patients the reason for exclusion was an alternate final diagnosis [osteomyelitis (three); gout (two); deep venous thrombosis (one)]. One patient was subsequently excluded because he was recognized later to be seventeen years of age at the time of enrollment. One patient was inadvertently enrolled into the study on two separate occasions and as such only the first presentation was analyzed.

The characteristics of the 95 analyzed patients and their infections are summarized in Table 2. The patients were most commonly middle-aged men with uncomplicated cellulitis who were referred from the emergency department.

**Table 2:** Characteristics of 95 patients with ESTI included in the item reduction phase.

Characteristic	Number
Mean years of age (SD)	47.5 (14.3)
Male gender (%)	74 (77.9)
Referral location (%)	
Emergency Department	86 (90.5)
Other	9 (9.5)
Comorbidities (%)	
Diabetes Mellitus	10 (10.5)
Peripheral Vascular Disease	7 (7.3)
Immunosuppression	10 (10.5)
Animal Bites	5 (5.2)
Post-surgical	3 (3.2)
Primary diagnosis (%)	
Cellulitis	77 (81.1)
Septic bursitis	12 (12.6)
Septic tenosynovitis	3 (3.2)
Abscess	3 (3.2)
Location of ESTI (%)	
Upper extremity	36 (37.9)
Lower extremity	59 (62.1)
Median duration of symptoms at time of survey (interquartile range)	4 (2–7)
Median days of intravenous antibiotics (interquartile range)	4 (3–6)
Median days total antibiotics (interquartile range)	14 (11–16)

The overall importance of each of the items was calculated as frequency-importance-products and reported in Table 1.

### Final questionnaire preparation

The twenty highest ranked items identified in item reduction (Table 1) were grouped into domains and included in the final questionnaire. They were presented in a similar fashion as in the item reduction questionnaire. These twenty items represent each of the HRQL domains including symptoms (seven items), daily functioning (seven items), emotional functioning (five items), and social interactions (one item). The final questionnaire was pre-tested on a group of ten patients. After filling out the survey independently they reviewed it with one of the study investigators. No patient reported any difficulty in completing the questionnaire or interpreting the items.

### The ESTI Score

**Figure 1:**
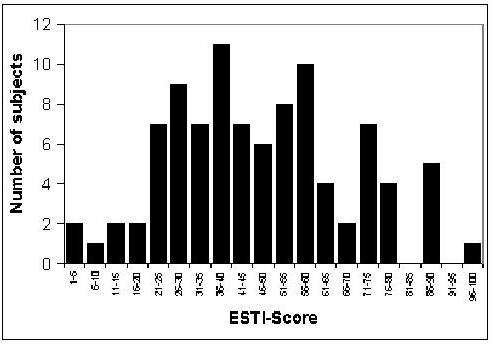
Distribution of ESTI-Scores calculated *post hoc *among 95 patients included in the item reduction phase.

The final ESTI-Score for a patient was calculated by adding their scores for each item. The minimum and maximum possible scores are zero and one hundred respectively. The ESTI-Score was *post hoc *calculated for each of the patients in the item reduction cohort. The mean ESTI-Score was 47.2 ± 21.1 points and their distribution is shown in Figure 1. No significant differences in mean scores were observed overall between men and women, cellulitis versus other ESTI, or upper versus lower extremity site of infection.

## Discussion

We report the development of the first HRQL indicator for use in patients with ESTI. This tool was developed using established methodological guidelines and included a diverse cohort of ambulatory adult patients with a variety of soft tissue infections. It incorporated items representing multiple areas of quality of life impairment including physical symptoms, activities of daily living, emotional and social functioning. When pre-tested on another group of patients the final questionnaire was found to be straightforward and easy to independently complete in less than 10 minutes. In a *post hoc *analysis, of our patient cohort, the ESTI-Score produced a broad range of near-normally distributed values, potentially reflecting the range of severity of ESTI in our patients.

Clinical trials of ESTI reported to date have largely used a variety of disease-oriented outcome measures. Often a categorical assessment such as "cure" or "failure", "resolution of infection", or "improvement" is assigned by a study investigator based on a non-standardized clinical assessment [14-22]. Another approach has been to use "microbiological eradication" or "microbiological persistence" as an outcome measure [16,18-20]. The utility of such an outcome measure is hampered by the fact that the majority of soft tissue infections are culture negative or will rapidly become culture negative even if sub-optimally managed [23,24]. Others have attempted to quantify improvement based on measurements of areas of inflammation although this has not been validated and may not be closely related to patients' symptoms [25]. While patients' subjective experiences are often included in a composite clinical assessment by a physician or study investigator, the use of a HRQL measure allows actual quantification of these experiences and the effect of treatment.

Generic health measures such as the Medical Outcomes Study 36-Item Short-Form Health Survey (SF-36) [26], and the Nottingham Health Profile (NHP) [27], have been developed to measure quality of life in a broad range of illnesses. They may not, however, be able to detect the subtle effects on quality of life specific to a particular disease. Specific HRQL scales have been developed and validated for other diseases and have been used as outcome measures in clinical trials [4-8]. Tools, such as the "Skindex," have been developed to evaluate the HRQL in chronic dermatologic conditions but are not applicable to acute soft tissue infections [9,10]. The ESTI-Score represents the first standardized clinical instrument to measure the areas of HRQL impairment important to patients with ESTI.

As a result of the ESTI-Score being developed using a cohort of patients, it does have face and content validity. However, there are a number of limitations of the current study. First, it requires further prospective assessment in another cohort of ESTI patients prior to being used as a primary outcome measure in clinical trials. Areas to be assessed include its reproducibility in patients with a stable disease state, its responsiveness to changes in infection severity, and its relationship to other markers of disease severity. Second, the ESTI-Score was derived from a group of North American adult patients receiving parenteral antibiotics at a single outpatient parenteral therapy clinic. Patients with mild disease were excluded based on the requirement for at least two days of parenteral therapy and only English speaking patients were studied. Whether it will be generalizable to adult patients in other outpatient settings in other regions remains to be seen. Third, while ESTI may have subsequent chronic impairment on quality of life, they typically are acute conditions that resolve over weeks or months and in most cases will not have a major effect on an individual's quality of life during their lifetime. The ESTI-Score may therefore represent more of an acute comprehensive disease severity index than a traditional measure of quality of life.

## Conclusion

We report the development of the ESTI-Score that is designed to quantify patients subjective experiences associated with having an ESTI. Future studies are needed to validate this HRQL instrument in another cohort of adult ESTI patients so that it may be used as an important outcome measure in future clinical trials evaluating management strategies for ESTI.

## Abbreviations

Extremity Soft Tissue Infections (ESTI)

Frequency importance product (FIP)

Health-Related Quality of Life Questionnaire (HRQL)

Home Parenteral Therapy Program (HPTP)

Interquartile range (IQR)

Medical Outcomes Study 36-Item Short-Form Health Survey (SF-36)

Nottingham Health Profile (NHP)

Soft tissue infections (STI)

## Competing interests

None of the authors have personal, professional, or financial conflicts of interest that would influence the conduct or reporting of this study.

## Authors' contributions

AJS collected data, analyzed results, and drafted the manuscript. KL conceived and designed the study, enrolled patients, analyzed data, and contributed to the manuscript drafting. RRR, MWM, JMG, DN, and TJL enrolled patients and collected data. All authors contributed to revision of and approval of the final manuscript.

## Pre-publication history

The pre-publication history for this paper can be accessed here:


